# Comment on ‘Renal failure suppresses muscle irisin expression, and irisin blunts cortical bone loss in mice’ by Kawao *et al*.

**DOI:** 10.1002/jcsm.13020

**Published:** 2022-06-06

**Authors:** Jing‐Fu Bao, Pan‐Pan Hu, Aiqing Li

**Affiliations:** ^1^ State Key Laboratory of Organ Failure Research, National Clinical Research Center for Kidney Disease, Nanfang Hospital Southern Medical University Guangzhou China; ^2^ Guangdong Provincial Key Laboratory of Renal Failure Research Guangzhou Regenerative Medicine and Health Guangdong Laboratory Guangzhou China

Recently, Kawao *et al*.^1^ reported their interesting work, which revealed that chronic kidney disease (CKD) is able to suppress muscular FNDC‐5 expression, and this is closely related to decreased bone mineral density. In addition, they also observed that exogenous irisin administration mitigates CKD‐induced reductions in cortical bone mineral density, but without affecting muscle mass. Nevertheless, there are some concerns about CKD model in this study which should be noted.

CKD is a state of progressive decline in renal function, as indicated by gradually increased serum creatinine, urea nitrogen, and renal fibrosis. In a previous study, C57BL/6J mice were considered to resist 5/6 nephrectomy‐induced CKD, which was presented as no hypertension, less glomerular damage, and renal fibrosis even 16 weeks after 5/6 nephrectomy; instead, 129S3 and CD‐1 (ICR) mice exhibited prominent CKD after 5/6 nephrectomy.^2^ Thus, C57BL/6J mice are not suitable to establish CKD model. In addition, compared with male mice, female mice are less sensitive to 5/6 nephrectomy‐induced progressive renal injury,^3^ indicating a gender‐dependent response to 5/6 nephrectomy.

It is curious that Kawao *et al*.^1^ chose C57BL/6J rather than other susceptible mouse strains and female rather than male mice to establish CKD model in this study. Although they reported increased serum creatinine and urea nitrogen in nephrectomized female C57BL/6J mice, they did not provide pathological features of these mice. Notably, they did not find altered expression of protein degradation‐related genes in muscular tissues of nephrectomized female mice, while this can be observed in 5/6 nephrectomized ICR mice.^4^ Hence, it is unclear whether they obtained a successful CKD model in this study, despite they observed decreased bone mineral density. Based on these, we wonder if these results would reflect the true effects of irisin in CKD‐related sarcopenia and bone disease.

Despite our doubts, we were so impressed that the conception of the study conducted by the authors may provide a potential therapeutic strategy to treat the CKD‐mineral bone disorder and sarcopenia in CKD patients.

## Conflict of interest

The authors declare that they have no conflict of interests.
